# Institutional Notes and News

**Published:** 1910-04-16

**Authors:** 


### Institutional Notes and News.

**Ear and Throat Hospital, Birmingham.**

No fewer than 42.6 per cent. of the in-patients received last year at the Birmingham and Midland Ear and Throat Hospital came from beyond the city and its suburbs. Even of the new out-patients 32.03 per cent. were outlandish persons in the strictest sense of the word. This illustrates the fact that in attempting to fix the locality of their patients and to work that for subscriptions special hospitals are at a disadvantage with those which offer less exclusive treatment. Special hospitals invite the whole world of special sufferers, while the general hospital can confine its treatment to a special locality. In the face of this difficulty the Midland Ear and Throat Hospital may congratulate itself on having no greater debt to complain of than £462 on its year's work. Mr. J. Milliken Smith's speech at the annual meeting seemed to show this feeling, by pointing out that such a debt is not exceptional; indeed, many institutions owe far more. Mr. James Whitfield, however, reminded the meeting that of the £8,000 spent on building with the proceeds of legacies £5,500 remained unsubscribed.

**Walsall Hospital, Wolverhampton.**

At the end of 1908 the Walsall Hospital decided to adopt a plan by which not more than fifty occupied beds should be maintained. The result of this has been apparently that 137 in-patients fewer than in the previous year were admitted to treatment. The out-patient department also is recorded to have had fewer attendances; although, as the annual report states that the effects of medical inspection of school children are still very heavy, it is not absolutely clear whether their attendances have been included. The hospital received £3,350, and spent £4,073, and this discrepancy has caused the hospital's total liability for maintenance to amount to £2,049. The ordinary income, unfortunately, was less by £108 than in the previous year. Mr. E. J. Cotterell remarked that it was not an altogether satisfactory report, but that the executive committee was determined to continue the policy of fifty beds already noticed. It should be added that the hospital was built to hold seventy beds, and that in 1907, the year before the fifty-bed policy was adopted, the average number of occupied beds was fifty too. The two wards now closed will not be re-opened till the money comes in to pay for their support.

**Glasgow Maternity Hospital.**

Dr. Robert Gourlay took the chair at the Glasgow Maternity and Women's Hospital annual meeting. He drew attention in his speech to one or two points of new departure in the hospital's policy, the principal of which is the clause under the new constitution which arranges for the placing of two working-men directors on the board. The wisdom, and indeed the simple fairness of this change, is clear when we remember that the wives of working-men form the majority of the patients. At Glasgow, as elsewhere, the value of ladies' work when ladies have agreed to undertake it was made prominent at this annual meeting also. Large alterations have been carried out, so large indeed that £20,000 is still wanted for the new buildings. The hospital and the branches treated 4,734 cases, but have also accumulated a debt of £3,263 on the current account. The best comment on this is perhaps that almost 1,000 more cases were treated last year than three years ago. Mr. Arthur Forbes, the Secretary, who submitted these figures, was succeeded by Dr. Robert Gourlay, who said that interest was growing through West Scotland in their institution, and that it was being increasingly attended as a training school by medical students.

**Chelsea Hospital for Women.**

The report of the Council of the Chelsea Hospital for Women was considered at the annual meeting of governors, over which Sir F. W. R. Fryer presided. The history of the past year may be succinctly stated. The in-patients numbered 801, an increase of eighty, and the hospital's income, owing to the absence of legacies—an absence of which nearly every hospital complains, was £1,000 less than the expenditure. In spite of the expenses which scientific sterilisation made to increase, the average weekly cost per patient has been reduced by 5s., and now is £2 0s. 11d. It is good to learn on the authority of Sir F. W. R. Fryer that £2,000 will soon be received from the Zunz bequest. A striking illustration of the advance made in surgery at the hospital is seen in the registrar's report of the operation for the removal of fibroid tumours called hysterectomy. Ten years ago twenty-three such operations were performed during the year, and two deaths resulted. Last year there were 113 of these operations, and no deaths. The rebuilding of the out-patient department, and over it of a nurses' home, is a pressing duty. The Convalescent Home is doing splendid work, and Mrs. George Hilditch has generously promised to give £100 for a garden shelter there in memory of the keen interest the late Mr. Hilditch had always taken in the Home.

**Annual Meeting at Worcester.**

Our readers will appreciate a note on the annual meeting of the Worcester General Infirmary in view of the Report which we publish elsewhere. During 1909 the in-patients numbered 1,268, a decrease of 71, while the out-patients numbered 6,596, an increase of 95. The daily average number of in-patients was 113.4, and the cost per bed £52 16s. 7¼d. The subscriptions have gone up, as also the Hospital Sunday Fund collection, and these total between them £1,847 odd. The hospital received £2,227 in the form of legacies, and the income for the year, excluding legacies, was £4,765. The expenditure for the year was £8,261, and a consequent deficiency exists of £3,496. Under the governor's sanction, given at the preceding meeting, £1,105 L.N.W.R. Three per Cent. Perpetual Debenture stock, and £1,000 N.E.R. Four per Cent. Preference stock, were sold, realising together £2,070. "The state of the finances of the infirmary," the annual report stated, "pointed to the necessity of further sales of invested stock during the current year." The Rev. R. N. Kane protested that the report would be misleading if it did not make clear that the deficiency of £3,496 was reduced in fact by their legacies (which amounted to £2,227), for all but a ten-pound note of these were actually carried to the current account. The report was adopted, and Mr. F. J. A. Wood's proposal that authority be given the trustees to sell out the funded property of the Infirmary, as may be required from time to time, sufficient to produce a sum not exceeding £3,000 to meet current expenses, was agreed to. A discussion took place on the way to detect abuse and to increase economy, in the course of which Mr. Leicester's suggestion that a small committee should be appointed to examine the financial position of the Infirmary was not adopted.

